# Hydroponic Nutrient Solution Temperature Impacts Tulane Virus Persistence over Time

**DOI:** 10.1007/s12560-024-09583-6

**Published:** 2024-02-27

**Authors:** Gayatri R. Dhulappanavar, Kristen E. Gibson

**Affiliations:** Department of Food Science, Center for Food Safety, University of Arkansas System Division of Agriculture, 1371 West Altheimer Dr., Fayetteville, AR 72704 USA

**Keywords:** Human norovirus, Surrogate, Hydroponic, Nutrient solution, Lettuce, Controlled environment agriculture (CEA)

## Abstract

Controlled environment agriculture (CEA), or indoor agriculture, encompasses non-traditional farming methods that occur inside climate-controlled structures (e.g., greenhouses, warehouses, high tunnels) allowing for year-round production of fresh produce such as leaf lettuce. However, recent outbreaks and recalls associated with hydroponically grown lettuce contaminated with human pathogens have raised concerns. Few studies exist on the food safety risks during hydroponic cultivation of leaf lettuce; thus, it is important to identify contributing risk factors and potential mitigation strategies to prevent foodborne transmission via hydroponically grown produce. In this study, the concentration of infectious Tulane virus (TV), a human norovirus surrogate, in hydroponic nutrient solution at 15 °C, 25 °C, 30 °C, and 37 °C was determined over a duration of 21 days to mimic the time from seedling to mature lettuce. The mean log PFU reduction for TV was 0.86, 1.80, 2.87, and ≥ 3.77 log_10_ at 15 °C, 25 °C, 30 °C, and 37 °C, respectively, at the end of the 21-day period. Similarly, average decimal reduction values (*D*-values) of TV at 15 °C, 25 °C, 30 °C, and 37 °C were 48.0, 11.3, 8.57, and 7.02 days, respectively. This study aids in the (i) identification of possible food safety risks associated with hydroponic systems specifically related to nutrient solution temperature and (ii) generation of data to perform risk assessments within CEA leaf lettuce operations to inform risk management strategies for the reduction of foodborne outbreaks, fresh produce recalls, and economic losses.

## Introduction

Controlled environment agriculture (CEA), or indoor agriculture, encompasses non-traditional farming methods that occur inside climate-controlled structures (e.g., greenhouses, warehouses, high tunnels) allowing for year-round production of fresh produce including tomatoes, cucumbers, and leafy greens (Sharma et al., 2018). Within these protected environments, deep water culture systems, wick method, aeroponics, and nutrient film technique are being used for hydroponic cultivation of fresh produce such as leafy greens (Riggio et al., 2019). During CEA production, plant roots remain immersed in a solution containing essential micro- and macro-nutrients throughout the cultivation period to aid in growth and development. Although CEA is often viewed as a way to mitigate microbial risks that are present within field-based produce production, the 2021 outbreak (McClure et al., 2023) and 2023 recall (USFDA, 2023) of CEA-grown leafy greens due to microbial contamination have highlighted the need to characterize microbial risks within hydroponic cultivation including those related to foodborne viruses such as human norovirus (HuNoV).

Human noroviruses are frequently associated with leafy greens outbreaks in the U.S. and globally (Callejón et al., 2015; Herman et al., 2015). The primary transmission route for HuNoV is fecal–oral via direct contact (person-to-person) followed by ingestion of contaminated food and water (Hirneisen & Kniel, 2013; Wu et al., 2023). Indeed, field workers and food handlers are most often implicated as the contamination source of leafy greens during field-based production and at the point of sale, respectively (Kokkinos et al., 2015). However, the risk of HuNoV contamination during hydroponic production of leafy greens has not been well characterized. Riggio and co-authors (2019) reviewed the risk of pathogen internalization within leafy greens during lab-scale cultivation experiments and described four studies related to foodborne viruses including HuNoV and its cultivable, surrogate viruses, i.e., Tulane virus (TV) and murine norovirus (MNV). Human norovirus, TV, and MNV were reported to internalize within the edible portion of romaine lettuce within 1 day after direct inoculation of the nutrient solution and persist for an additional 14 days during lab-scale cultivation (DiCaprio et al., 2012). Wang and Kniel, (2016) also reported the internalization of MNV within the edible portion of microgreens cultivated using nutrient film technique along with MNV persistence in the microgreens at approximately 1.5 logs per sample from day 8 (day of inoculation) to day 12 (harvest day). Importantly, the study authors noted high concentrations of infectious virus remained in the recirculating nutrient solution for the duration of the experiment (Wang & Kniel, 2016). Importantly, no studies have investigated infectious virus persistence within deep water culture systems used for leaf lettuce production.

The role of water as a contamination source during field-based fresh produce production is well-documented (Alegbeleye et al., 2018; Gurtler & Gibson, 2022; Uyttendaele et al., 2015). In addition, the persistence of HuNoV and its surrogate viruses within a variety of water sources for prolonged periods of time have been reported (Anderson-Coughlin et al., 2023; Desdouits et al., 2022; Seitz et al., 2011). However, specific investigations on the persistence of infectious virus particles within nutrient solution under commercially relevant formulations and temperatures have not been reported. The limited number of previous studies on viral pathogens and hydroponic leafy greens production have performed experiments under a single temperature typically set around 20–22 °C, yet nutrient solution temperatures can fluctuate within greenhouse operations due to diurnal and seasonal variations potentially impacting virus persistence.

In the present study, the authors aimed to determine the persistence of infectious TV—a cultivable HuNoV surrogate—inoculated in modified Hoagland’s nutrient solution subjected to the wide-range of temperatures (10–40 °C) observed during production of leaf lettuce under greenhouse conditions over a 21-day period (i.e., to mimic leaf lettuce growth period from seedling to mature plant).

## Materials and Methods

### Mammalian Cell Cultivation

The LLC-MK2 cells (ATCC CCL-7; American Type Culture Collection, Manassas, VA) were grown in M199 medium (Cytiva, Marlborough, MA) supplemented with 10% Fetal Bovine Serum (FBS, Cytiva), 1% Penicillin–Streptomycin (100 U/mL, 100 μg/mL; Cytiva), and 1% amphotericin B (250 μg/mL; Corning, VA) at 37 °C and 5% CO_2_. Tulane virus was provided by Dr. Jason Jiang at Cincinnati Children’s Hospital Medical Center in Cincinnati, OH.

### Tulane Virus Production and Quantification

Tulane virus production and quantification was completed as previously described by Arthur and Gibson (2015a, b). Tulane virus was generously provided by Dr. Jason Jiang (Cincinnati Children’s Hospital Medical Center, Cincinnati, Ohio). Briefly, MK2 cells were inoculated with TV at a multiplicity of infection of 0.1. The flask with inoculated MK2 cells was rocked under 37 °C, 5% CO_2_ for 1 h followed by the addition of 20 mL of maintenance medium (2% FBS supplemented Opti-MEM) (Gibco™ Thermo Fisher Scientific, Waltham, MA). Further, the infected cells were incubated for 48 h at 37 °C, 5% CO_2_ without rocking. At the end of incubation, the flask was tapped vigorously to detach all cells. Viruses were harvested by three times freeze–thaw (− 80 °C and 37 °C) to release the viruses from the cells. The lysed cells were pelleted by centrifugation at 3000 × *g*, 4 °C for 15 min. The virus supernatant was then filtered through a 0.45 μm pore size bottle top vacuum filter (Corning Inc., Corning, NY) (Deng & Gibson, 2023). No additional methods were applied to ensure complete removal of spent maintenance medium or to prevent virus aggregates prior to inoculation of the nutrient solution (see “Inoculation of Nutrient Solution”).

MK2 cells were seeded in 6 well plates at a concentration of 2 × 10^5^ cell/well 24 h prior to performing the plaque assay. After overnight incubation, 500 μL of TV stock were added per well in duplicate. The plates were gently rocked for 1 h at 37 °C under 5% CO_2_. Samples were aspirated, and cell monolayers were covered with 2 mL overlay containing 3% low melting agarose (NuSieve® GTG Agarose; Lonza, Basel, Switzerland) and maintenance medium. The plates were further incubated at 37 °C for 5 days followed by staining with 2 mL of 5% neutral red (Sigma-Aldrich, St Louis, USA) diluted in 1X phosphate buffered saline (PBS, pH 7.4). To visualize plaques, the plates were incubated for an additional 3 h at 37 °C under 5% CO_2_ without rocking. The average concentration of the TV stock utilized in the study was 6.99 × 10^6^ plaque forming units (PFU) per mL.

### Preparation of Nutrient Solution

A modified Hoagland’s nutrient solution was used for all experiments as described previously (Dhulappanavar & Gibson, 2023). Briefly, macronutrient concentrations (in g/L) present in the stock A solution included 208.75 calcium nitrate, 65 potassium nitrate, and 28.49 mono potassium phosphate, and the stock B solution contained 100.63 g/L magnesium sulfate and 27.50 g/L potassium chloride (Haifa Group, Altamonte Springs, FL). Micronutrient concentrations in the stock C solution contained 3 g/L ferric-ethylenediaminetetraacetic acid (Fe-EDTA), 1.5 g/L manganese ethylenediaminetetraacetic acid (Mn-EDTA), 0.5 g/L boric acid, 0.38 g/L copper sulfate, 0.28 g/L zinc sulfate, and 0.05 g/L sodium ammonium molybdate (JR Peters, Inc, Allenstown, PA). Six hundred milliliters of hydroponic nutrient solution were prepared by mixing 3 mL of each stock solution (stock solution A, B and C) containing macronutrients and micronutrients in 1:1 ratio, 0.12 mL sodium thiosulphate (Sigma Chemical Co., St. Louis, MO) stock solution (12.5 ppm), and 590.88 mL of tap water. The initial pH of the prepared hydroponic nutrient solution was adjusted to within the range of 5.51—5.54 using 1N sulfuric acid.

### Inoculation of Nutrient Solution

Single 500 mL glass bottles containing 300 mL nutrient solution were each inoculated with 900 µL of TV stock solution (or ~ 6.24 log_10_ PFU total). The final TV concentration was approximately 4 log_10_ PFU per milliliter of nutrient solution. The bottle with inoculated nutrient solution and another bottle containing 300 mL nutrient solution only (negative control) were placed in a shaking incubator. The incubator was maintained at 15, 25, 30, or 37 °C for 21 days with shaking at 50 rpm.

### Sample Collection and TV Quantification

Post inoculation, 30 mL of sample was collected from each bottle on day 0, 1, 3, 5, 7, 14, and 21. At each time point, 5 mL of the collected sample was filtered using a 0.22 µm syringe filter (VWR EZFlow® Syringe Filter, Hydrophilic PVDF, Sterile, Foxx Life Sciences) to eliminate any bacteria that may be present. TV inoculated nutrient solution samples were used for serial dilution, and 500 μL of each dilution (or in some cases, undilute sample) was added in duplicate to 6-well plates containing 80–90% confluent MK2 cell monolayer. Samples without TV (negative control) were added to a separate 6-well plate. Plaque assays were performed as described previously in “Tulane Virus Production And Quantification”. The results obtained were expressed in PFU per mL. The limit of detection (LOD) for the assay is 1 PFU/mL.

Preliminary studies were conducted to determine any cytotoxic effect of the nutrient solution on the MK2 cells during the plaque assay. Briefly, nutrient solution at undilute, twofold, fivefold, tenfold, and 100-fold dilutions were applied to 6-well plates with MK2 cells without TV as well as spiked with a known amount of TV to determine impact on the plaque assay. Results indicated no cytotoxic effect on the MK-2 cells, and no impact on virus plaque formation and the expected number of plaques.

### Data Analysis

In this study, three trials were performed at each temperature (15, 25, 30, or 37 °C), and technical duplicates were completed. A completely randomized block design with a split-split plot was used for experimental design. Before data analysis, values below the LOD were assigned a value of one-half the LOD, or 0.5 PFU/mL. All data were log-transformed by taking the log_10_(PFU + 1). More specifically, the LOD was reported as 0.5 PFU, and thus, resulting in a negative log value. By adding 1 to the number of PFU for each data point prior to log transformation, all values are positive without impacting the statistical analysis (Baker et al., 2021). Outliers were detected using the interquartile range where data points below the 10th percentile and above the 90th percentile were deemed outliers and removed from the dataset.

A mixed model was used to determine the effect of temperature and time (day) on TV concentration (log_10_ [PFU + 1]/mL). Least squares mean estimates were compared with Tukey’s HSD test, and *P* < 0.05 was considered statistically significant. In addition, decimal reduction values (*D*-values) were calculated using the slope from linear regressions by plotting each data point (log_10_ [PFU + 1]/mL) from each temperature. *D*-values indicate one log (90%) virus reduction (in days) in hydroponic nutrient solution for each temperature. All data were analyzed using JMP® Pro 17 (SAS Institute, Inc., Cary, NC, USA) software.

## Results

### Hydroponic Nutrient Solution Temperature Impact on Infectious TV

Least squares means and 95% confidence intervals for TV across each temperature and time are provided in Table 1. Statistical analysis indicated that a 2-way interaction effect between temperature and sampling day had a significant effect on TV persistence in nutrient solution (*P* < 0.0001), thus interpretations cannot be made for main effects. At 15 °C and 25 °C, there was no significant effect (*P* = 0.7991 and *P* = 0.0594, respectively) on TV persistence in hydroponic nutrient solution from the initial day of sampling (day 0) to the final day of sampling (day 21). However, 30 °C and 37 °C had significant effects (*P* < 0.0001 at both temperatures) on infectious TV concentrations in nutrient solution from the initial to final day of sampling. The average pH and electrical conductivity of inoculated and control nutrient solution were 5.89 ± 0.29 and 2.25 ± 0.15 mS/cm and 5.57 ± 0.45 and 2.27 ± 0.15 mS/cm, respectively.

**Table 1:** Least squares mean [95% CI] of infectious TV in nutrient solution at different temperatures across a 21-day period for experiments performed in triplicate

Day	15 °C	25 °C	30 °C	37 °C
0	4.05 [3.54, 4.56]	4.02 [3.51, 4.53]	4.11 [3.60, 4.62]	3.95 [3.44, 4.46]
1	3.85 [3.34, 4.36]	3.70 [3.08, 4.32]	3.19 [2.68, 3.70]	2.29 [1.78, 2.80]
3	3.99 [3.48, 4.50]	3.60 [3.09, 4.11]	3.06 [2.55, 3.57]	1.76 [1.25, 2.27]
5	3.72 [3.21, 4.23]	3.18 [2.67, 3.69]	3.15 [2.64, 3.66]	1.32 [0.81, 1.83]
7	3.67 [3.16, 4.18]	2.50 [1.99, 3.01]	2.97 [2.46, 3.48]	0.18*
14	3.63 [3.12, 4.13]	3.00 [2.37, 3.62]	1.70 [1.19, 2.21]	0.18*
21	3.19 [2.68, 3.70]	2.47 [2.85, 3.09]	1.24 [0.73, 1.75]	0.18*

^*^All samples below the LOD thus least squares means [95% CI] were not calculated

The limit of detection (LOD) is 1 PFU/mL. Samples below the LOD were assigned a value of one-half the LOD (0.5 PFU/mL) which, after data preprocessing (i.e., PFU + 1), is 0.18 log ([PFU + 1]/mL). The dataset was then evaluated for outliers using the interquartile range where data points below the 10th percentile and above the 90th percentile were deemed outliers, and when detected, these values were removed. One outlier was detected each on Day 14 and 21 at 25 °C within one experimental replication

### Estimated *D*-values (in Days) for TV in Hydroponic Nutrient Solution by Temperature

**Fig. 1:**
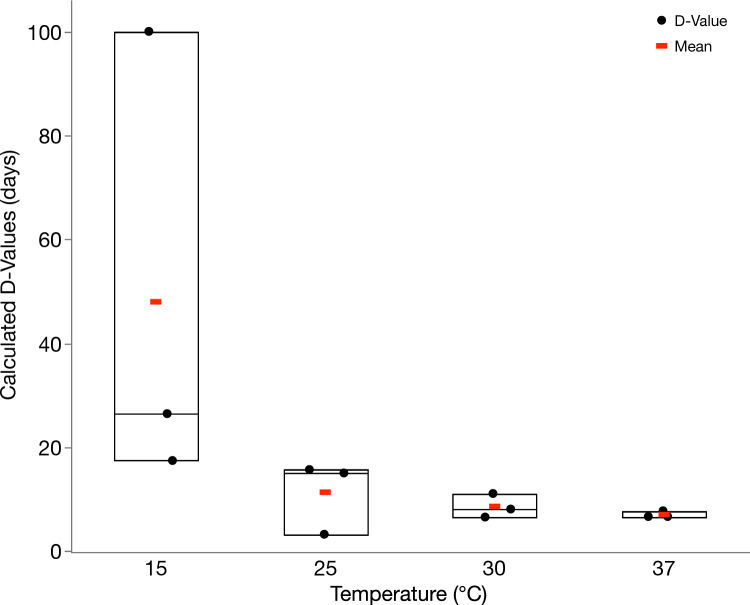
Calculated decimal reduction values (D-values) in days for the persistence of Tulane virus in nutrient solution for a duration of 21 days (*n* = 3). Points represent the calculated D-value for each experimental replication. Outliers were omitted from the dataset as described in “Data Analysis” and included day 14 and 21 for 25 °C for one experimental replication. Values below the LOD were assigned a value of one-half the LOD, or 0.5 PFU/mL. All data were log-transformed by taking the log_10_(PFU + 1). Data below the LOD were assigned a log_10_ value of 0.18

Slopes from linear regression equations were used to calculate the *D*-values, or time (in days) to attain a 1 log_10_ reduction of TV in one milliliter of hydroponic nutrient solution, at different temperatures. Lack of fit tests were completed and indicated a statistically significant (*P* = 0.0358) lack-of-fit F statistic at 25 °C. However, given the moderate significance in the lack of fit at 25 °C, *D*-values were still calculated using the linear fit model. The calculated R-squared values based on the fit lines for 15, 25, 30, and 37 °C were 0.41, 0.49, 0.72, and 0.80, respectively. Figure 1 displays the mean estimated *D*-values at 15, 25, 30, and 37 °C which were determined to be 48.0, 11.3, 8.57, and 7.02 days, respectively, indicating that TV may persist in hydroponic nutrient solution for a prolonged duration at lower temperature (15 °C) compared to higher temperature (37 °C).

## Discussion

Overall, the present study indicates the significant role of temperature in TV persistence in modified Hoagland’s nutrient solution. A few previous studies have characterized the persistence of viruses of public health concern, including HuNoV and its surrogate viruses, within nutrient solutions for hydroponic production of produce. Carducci and co-authors (2015) described the persistence of the enterovirus, Coxsackievirus B2, within a lab-scale hydroponic system for the cultivation of lettuce. The study authors inoculated 0.5 M sterile Hoagland’s solution with Coxsackievirus B2 for a final concentration of approximately 9.3 log_10_ genomic copies per liter (gc/L). While the temperature of the nutrient solution was not reported, lettuce cultivation experiments were controlled at 23 °C and 70% relative humidity over a 7-day period under a fluorescent light cycle of 12 h light and 12 h dark (Carducci et al., 2015). The authors observed a 5 log_10_ gc/L reduction in Coxsackievirus B2 within the nutrient solution by day 3, and the virus was below the limit of detection by day 4. This rapid decline in enterovirus persistence differs from the TV reduction of ~ 0.5 log_10_ PFU/mL observed at a similar temperature (25 °C) on day 3 in the present study (see Table 1). Indeed, previous reports have indicated greater inactivation of viruses in water (i.e., groundwater) once temperatures exceed 20 °C (John & Rose, 2005). This difference in inactivation rate also highlights the variation that would be expected when comparing viruses belonging to different genera such enteroviruses and caliciviruses.

The persistence of the HuNoV surrogate, MNV, was also characterized within nutrient solution used for the production of kale and mustard microgreens (Wang & Kniel, 2016). Briefly, recirculating nutrient solution was inoculated with ~ 3.5 log_10_ PFU/mL MNV on day 8 in systems with and without microgreens, and the temperature of the greenhouse was maintained at an average of 22.3 °C. For samples collected from systems without microgreens (i.e., only recirculating nutrient solution), MNV was detected in the nutrient solution at 2.73 log_10_ PFU/mL on day 12. The ~ 0.8 log_10_ PFU/mL reduction of MNV in nutrient solution over 4 days at ~ 22 °C is similar to the TV reduction of ~ 0.5 log_10_ PFU/mL at 25 °C reported on day 3 in the present study (see Table 1). Meanwhile, Dicaprio et al. (2012) reported a 1 to 2 log_10_ PFU/mL reduction for both MNV and TV along with a 1 to 2 log_10_ gc/mL reduction in HuNoV (GII.4) in nutrient solution by day 4 during romaine lettuce cultivation at 20 °C and 40% relative humidity. Notably, DiCaprio and coauthors (2012) did not include a no plant control, thus the presence of lettuce likely impacted virus reduction in the nutrient solution over time. Indeed, TV was shown to associate with the plant roots and further internalized into the roots, shoots, and leaves over time (Dicaprio et al., 2012). In addition, the nutrient solution was described as hydroponic feed water supplemented with a nutrient solution containing nitrogen, phosphorus, and potassium with no further details provided such as pH or relevance to commercial formulations.

The persistence of infectious virus in nutrient solution at temperatures outside of the 20 to 25 °C range has not been reported to our knowledge. To understand the significance of results in the present study, studies on viral persistence in water under different temperatures are considered. Wu and coauthors (2023) determined the persistence of TV and MNV within three water sources (irrigation tailwater, groundwater, ultrapure water) held at 11, 19, and 24 °C for 28 days. The authors inoculated the water sources with 3.6 to 4 log_10_ PFU/mL TV and 4.1 to 5 log_10_ PFU/mL MNV and observed no significant difference in virus infectivity based on the type of water source or temperature for the 28-day study period (Wu et al., 2023). However, there were significant differences in virus concentration between day 0 and day 28 within each water type and temperature combination. A maximum reduction of 1.5 log_10_ PFU/mL was reported for the duration of the study with slightly greater reductions seen at 11 °C compared to 19 and 24 °C. These results differ from the persistence of TV in modified Hoagland’s nutrient solution observed in the present study with less than 1 log_10_ PFU/mL reduction at 15 °C and ~ 2.4 log_10_ PFU/mL reduction at 25 °C after 21 days. The reason for the observed differences at 25 °C across studies are not clear, though could be due to the pH of the nutrient solution (pH 5.5) compared to the pH of the water sources, i.e., 7.36 to 7.76 across all water types, in Wu et al. (2023). To our knowledge, no published studies have investigated the impact of prolonged (> 150 min) exposure of TV within water (or other solutions) at a pH outside of the neutral range (pH 6.5 to 8), although TV inactivation would not be expected at pH 5.5 as studies have shown little impact on infectivity at a pH ranging from 2 to 9 (Arthur & Gibson, 2015b, 2016; Bai, 2020; Cromeans et al., 2014; Hirneisen & Kniel, 2013; Wu et al., 2023).

Previous research has shown that virus aggregation—relevant to both virus persistence and accurate quantification—is dependent on pH along with the types of salts in solution and, most importantly, virus type (Gerba & Betancourt, 2017). Bai (2020) and Fuzawa et al. (2020) observed the pH-dependent aggregation of TV with an increase in aggregate diameter as the pH decreased from 7 to 3. Thus, the greater log_10_ reduction reported in the present study for TV in nutrient solution at 25 °C compared to Wu et al. (2023) could have been the result of greater TV aggregation leading to inaccurate quantification. Indeed, Gassilloud and Gantzer reported that 8 to 26% of the observed decrease of poliovirus titer in groundwater after 20 days was due to virus aggregation (Gassilloud & Gantzer, 2005). Regarding the role of salts in TV aggregation, minimal information is available, although divalent cations (e.g., Ca^2+^, Mg^2+^, Fe^2+^) are reported to have a stronger effect on aggregation compared to monovalent cations (e.g., Na^+^, K^+^, Cu^+^) (Gerba & Betancourt, 2017). Each of the aforementioned salts are present in the modified Hoagland’s nutrient solution and could impact TV aggregation and downstream quantification.

Hirneisen and Kniel (2013) investigated the persistence of TV and MNV in tap water held at 4 and 20 °C over a 30-day period. The authors reported that both viruses were significantly reduced (> 5 log_10_ PFU/mL) by day 25 at 20 °C. This differs from the present study where TV remained relatively stable at 15 °C for 21 days and only decreased by 2.4 log_10_ PFU/mL at 25 °C. However, these differences may be explained by the use of dechlorinated tap water in the present study to prepare the modified Hoagland’s nutrient solution while Hirneisen and Kniel (2013) did not dechlorinate the tap water prior to virus inoculation. The World Health Organization (2014) recommends residual chlorine concentrations in drinking water to be within the range of 0.2 and 5 ppm, preferably between 0.4 and 0.6 ppm at the tap. Although these chlorine concentrations are low, TV has been shown to be sensitive to chlorine treatment even at low concentrations (Hirneisen & Kniel, 2013; Tian et al., 2013). For instance, at 20 °C, TV was reduced by 1.33 ± 0.13 and 2.11 ± 0.62 log_10_ PFU/mL when exposed to 0.2 and 2 ppm chlorine for 5 min, respectively (Hirneisen & Kniel, 2013). Even still, the presence of residual chlorine does not fully explain the difference in TV inactivation rates across similar temperatures.

For temperatures greater than 25 °C, Arthur and Gibson (2015b) previously reported a *D*-*value* of 500 min (or 0.35 days) for TV held at 37 °C in PBS compared to 7.02 days estimated in the present study. These differences in TV inactivation rates at 37 °C are likely due primarily to the difference in study periods where Arthur and Gibson (2015b) collected samples over a 2-h period as opposed to multiple days, thus decreasing the accuracy of the estimated *D-value*. Tian and coauthors (2013) also reported no changes in TV concentration after a 30-min exposure to 37 °C. In general, research has repeatedly shown that human enteric viruses and their surrogates are relatively stable at temperatures ≤ 37 °C compared to those > 37 °C (Arthur & Gibson, 2015a).

The present study indicates that temperature can possibly impact the persistence of human norovirus (HuNoV) in hydroponic nutrient solution during production. Also, prolonged persistence of HuNoV in the nutrient solution at lower temperatures during hydroponic production could possibly increase the risk of internalization into the leafy greens. To further our knowledge regarding the effect of temperature on HuNoV during hydroponic production, future studies will focus persistence and internalization of TV within lettuce across multiple cultivars produced using recirculating deep water culture hydroponic systems within a greenhouse. Overall, it is evident that increased vigilance and improvised food safety guidelines are required to understand and mitigate potential food safety risks associated with hydroponic cultivation. This will aid in reducing foodborne outbreaks caused by human pathogens, fresh produce recalls, and economic losses.

## Data Availability

The data that support the findings of this study are available from the corresponding author upon reasonable request.
